# Phasor-FLIM and SHG imaging for quantitative analysis of lung cancer autofluorescence

**DOI:** 10.1016/j.csbj.2025.08.010

**Published:** 2025-08-12

**Authors:** Luca Pesce, Maria Giovanna Mastromarino, Greta Alì, Cristina Niccoli, Giuseppe Sancataldo, Marco Scotto, Giuseppe Vicidomini, Paolo Bianchini, Alberto Diaspro, Marco Lucchi, Nicola Belcari

**Affiliations:** a Center for Instrument Sharing of the University of Pisa (CISUP), Pisa, Italy; b University Hospital of Pisa, Division of Thoracic Surgery, Cardiac, Thoracic and Vascular Department, Pisa, Italy; c University of Pisa, Department of Surgical, Medical and Molecular Pathology and Critical Care Medicine, Pisa, Italy; d University Hospital of Pisa, Unit of Pathological Anatomy, Pisa, Italy; e University of Palermo, Department of Physics and Chemistry “Emilio Segrè”, Palermo, Italy; f Italian Institute of Technology (IIT), Genoa, Italy; g University of Genoa, Department of Physics, Genoa, Italy; h University of Pisa, Department of Physics “Enrico Fermi”, Pisa, Italy; i INFN Pisa, Italy

**Keywords:** Fluorescence Lifetime Imaging Microscopy, Phasor analysis, Second Harmonic Generation imaging, Autofluorescence signatures, Tumor microenvironment, Lunga cancer

## Abstract

Histopathology using hematoxylin and eosin (H&E) staining remains the gold standard for tumor diagnosis. However, extracting quantitative data from stained slides is challenging, limiting the ability to obtain objective biomarkers for disease progression. Tissue autofluorescence provides an alternative by exploiting endogenous fluorophores, such as collagen, elastin, and NAD(P)H, which provide optical signatures of tissue pathology. Fluorescence Lifetime Imaging Microscopy (FLIM), when combined with phasor-based analysis, enables quantitative, fit-free assessment of metabolic and structural changes in tissues, simplifying data interpretation and enhancing diagnostic accuracy. In this study, we applied a phasor-FLIM approach to systematically analyze two distinct histotypes of non-small cell lung cancer (NSCLC): adenocarcinoma (ADC) and squamous cell carcinoma (SQC). Our findings revealed significant elastin deposition (elastosis) in tumor tissues. By integrating FLIM with second harmonic generation (SHG) imaging, we characterized the fiber compositions in healthy versus tumor tissues, distinguishing between collagen and elastin autofluorescence signatures. This combined imaging strategy allowed for precise discrimination of tumor regions in unstained biopsy sections, demonstrating the potential of autofluorescence-based techniques for enhanced cancer diagnostics. These results highlight the advantages of FLIM and phasor analysis in providing quantitative insights into tumor microenvironments, facilitating the histopathological assessments in unstained tissue slices.

## Graphical Abstract

**Figure:**
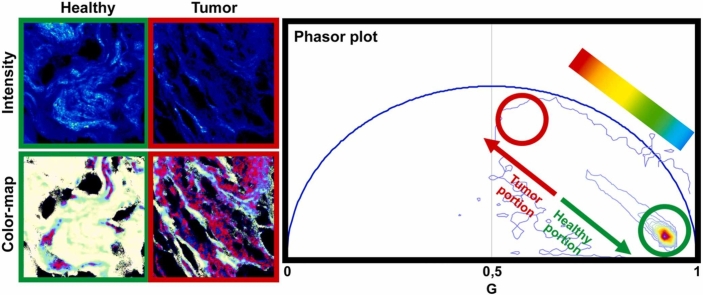

## 1 Introduction

The “gold standard” for diagnosing tumor progression involves generating and analyzing histopathology slides, typically stained with hematoxylin and eosin (H&E) [1], [2], [3]. This approach is widely used because it provides high contrast imaging under brightfield microscopy, allowing pathologists to identify morphological changes associated with the initiation and progression of tumors [4], [5], [6]. Analysis of these slides by trained pathologists offers highly accurate tumor staging and patient outcome predictions. However, extracting additional quantitative data from these tissue samples has remained a significant challenge. Such data could enhance pathologists’ ability to characterize and diagnose tumors more effectively. Introducing reproducible quantitative parameters into histopathology analysis could improve the accuracy of traditional, pattern-recognition-based examinations and provide objective biomarkers for disease progression [7].

While H&E-stained slides have historically been the focus of such investigations, an alternative approach involves examining unstained biological specimens using fluorescence microscopy [8], [9]. This is possible because cells and extracellular matrix contain endogenous fluorophores, which can be visualized and analyzed without the need of specific staining protocols.

Tissue autofluorescence arises from the intrinsic fluorophores present both inside and outside cells, such as collagen, elastin, pyridine nucleotides (i.e., nicotinamide adenine dinucleotide phosphate (NAD(P)H)), and flavins (i.e., flavin adenine dinucleotide (FAD)) [10], [11], [12], [13]. The fluorescence signatures of these substances, along with their spatial distributions, can provide valuable insights into the tissue’s physiological and disease state. By analysing the spectra of the intrinsic autofluorescence, as well as fluorescence lifetime, further information can be obtained [14], [15], [16].

Collagen and elastin are abundant in the extracellular matrix and can be used to investigate both healthy and pathological tissue conditions [17], [18]. Both molecules have been studied using various techniques, including immunohistochemistry, second harmonic generation (SHG) imaging, electron microscopy, and high-performance liquid chromatography (HPLC) combined with mass spectrometry [19], [20], [21], [22], [23]. Fluorescence-based methods have been particularly useful for characterizing collagen and distinguishing it from other tissue components [18]. Traditionally, the primary approach for investigating collagen has been staining excised tissues with picrosirius red [24]. While widely used, this technique is pathologist-dependent and cannot be automated. SHG imaging has also been extensively employed to visualize collagen fibrils. Due to the non-centrosymmetric structure of certain collagen fibers, SHG signals are generated without the need for labelling [21], [25], [26], [27].

Fluorescence Lifetime Imaging Microscopy (FLIM) has emerged as a powerful tool for studying cellular metabolism and its alterations during critical processes such as disease progression, differentiation, cell fate determination, inflammation, and cell division [28], [29], [30], [31], [32], [33]. Also, FLIM is combined with phasor-based graphical analysis technique, which simplify data interpretation [34], [35]. The phasor approach was first introduced by Digman and colleagues in 2008 [34], who demonstrated the use of trajectories in the phasor domain to analyze complex biological systems. Then, Stringari et al. applied the phasor method to study metabolic changes during stem cell differentiation [31]. Other applications, including the characterization of different collagen types and fibrosis conditions, were assessed using phasor-FLIM [36], [37]. Its fit-free capabilities make the phasor approach advantageous for FLIM image analysis, as it simplifies the process and avoids the challenges of traditional exponential fitting. Also, it provides a global, intuitive representation of the fluorescence decay curve for each pixel, making it easier to interpret complex data [35].

In recent years, the FLIM has gained traction in clinical and diagnostic applications [38], [39]. For example, it has been used for the pre-diagnosis of tumor pathology, as a screening tool for basal cell carcinoma, and for the detection of breast cancer [40], [41]. These advancements highlight the potential of FLIM and phasor analysis to provide comprehensive understanding into disease mechanisms and improve diagnostic accuracy. Different conditions have been characterized by exploiting tissue autofluorescence. For instance, Wang et al. [40] used a phasor-FLIM approach to systematically analyze cervical tissues from low- to high-risk lesions, while Conklin et al. [41] used fluorescence lifetime analysis to discriminate between normal and transformed mammary epithelium. In addition, lifetime measurements have been successfully used to investigate diseases affecting the brain, bones, and vasculature, as well as for drug evaluation [38].

In this study, we applied a phasor-FLIM approach to systematically analyze two distinct histotypes of non-small cell lung cancer (NSCLC): adenocarcinoma and squamous cell carcinoma. These tumors are the two most common subtypes of lung cancer (LC) representing 50–60 % and 20–30 % of total NSCLC cases, respectively [42]. Most of the tumors examined exhibited significant fiber deposition within the tumor microenvironment (TME), a process known as fibrosis [43], [44]. The deposition of fibers in the tissue is often mistakenly attributed to a single molecule, such as collagen [45]. However, this process is highly complex and involves multiple types of molecules, including collagen and elastin in lung. Indeed, the TME consists of cells, a fibrous macromolecular network, and the extracellular matrix's fluid phase. The extracellular matrix acts as a 3D scaffold, mainly composed of collagen types I and III for tensile strength, elastin for elasticity, and glycosaminoglycans (GAGs) in the non-fibrillar compartment [46], [47]. By combining SHG and FLIM-phasor analysis, we investigated the fiber type in both healthy and tumor tissues, highlighting key differences in unstained biopsy sections. This combined approach was used to identify the signature positions in the phasor plot corresponding to the autofluorescence of different fiber types from individual biopsies, enabling the discrimination between healthy and tumor regions in patient samples. Finally, we demonstrated that lung adenocarcinoma and squamous cell carcinoma were characterized by a high deposition of elastin, a pathological condition known as elastosis.

## 2 Methods

### 2.1 Participants and tissue samples

This study was conducted in accordance with the principles of the 1975 Helsinki Declaration and was approved by the local Ethics Committee. Each patient signed a written informed consent before surgical resection and data collection, and their use in clinical studies in anonymous form. Moreover, this study did not interfere with routine clinical practice.

**Fig. 1:**
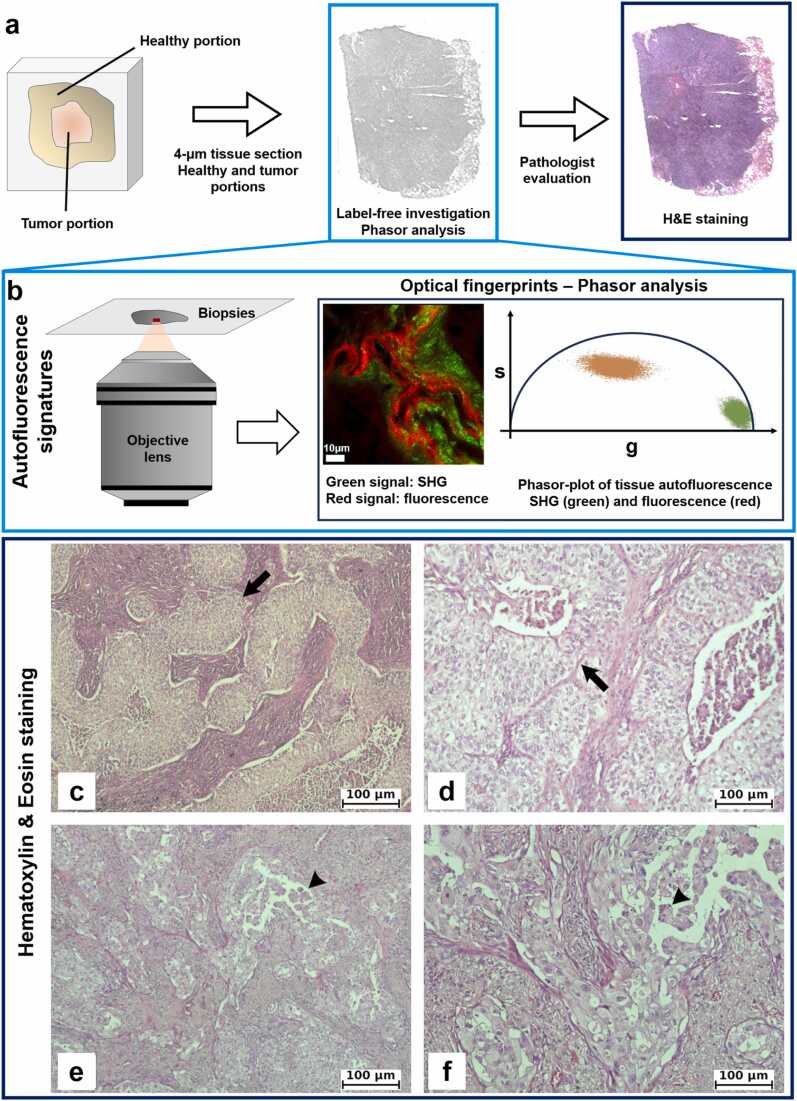
Experimental workflow for lung adenocarcinoma and squamous cell cancer. a) Experimental pipeline from biopsy extraction to H&E validation. Our method is positioned at the center of the pipeline, where unstained biopsies were imaged using an infrared laser. b) For both tumor and healthy regions, we investigated distinct optical signatures: NAD(P)H, elastin (visualized in orange), and SHG (in green), which were analysed using a phasor plot approach. Also, validation with H&E was performed. Histological characteristics of poorly differentiated squamous cell carcinoma (SQC1) and poorly differentiated adenocarcinoma (ADC1): c) and d) Squamous cell carcinoma showing infiltrative growth pattern with irregular tumor nests mixed with fibrosis. e) and f) Adenocarcinoma with solid tumor sheets (arrows) and micropapillary structures (arrowhead) with fibrous surrounding stroma.

Five cases of NSCLC, 2 squamous cell carcinomas (SQC) and 3 adenocarcinomas (ADC), were included in this study. The samples came from patients who had been submitted to surgical resection at the Unit of Thoracic Surgery of the University Hospital of Pisa. Histological diagnoses were performed and reviewed by expert pathologist (GA) according to the WHO 2021 histological and immunohistochemical criteria. SQC1 and SQC2 samples were poorly differentiated tumors. Regarding ADC samples, ADC1 and ADC2 were poorly differentiated and ADC3 moderately differentiated tumors, respectively. The samples were formalin fixed and paraffin embedded (FFPE). Then, tissue slices of 4-µm thickness were cut from each block and stained with H&E for histopathological examination. Histopathological characteristics of SQC1 and ADC1 samples are shown in Fig. 1.

### 2.2 H&E and DAPI & Eosin staining

Tissue sections of ∼4-μm thickness were stained with H&E following a standard protocol. Initially, the slides were deparaffinized in xylene for 6 min, followed by a sequential rehydration in graded ethanol solutions: 99 % ethanol for 3 min, 95 % ethanol for 3 min, and 70 % ethanol for another 3 min. After rehydration, the sections were stained with hematoxylin for 5 min, then rinsed under running tap water. To differentiate the hematoxylin staining, a brief immersion in hydrochloric acid alcohol (3 s) was performed, followed by an additional rinse in running tap water for 1 min and then in distilled water for another minute. Counterstaining was carried out using eosin Y for 3 min. The slides were then dehydrated through ascending ethanol concentrations: 95 % ethanol for 3 min, and three consecutive immersions in 99 % ethanol, each for 3 min. Finally, the sections were cleared in 100 % xylene before mounting.

For eosin and DAPI staining, sections underwent deparaffinization through three consecutive 15-minute immersions in xylene, followed by rehydration in a graded ethanol series: 99 %, 95 %, 70 %, and 50 % ethanol, each for 10 min. The sections were then stained with eosin for 5 min and briefly rinsed in 95 % ethanol for 3 min. After air-drying, the nuclei were counterstained with DAPI, and the slides were mounted with coverslips.

### 2.3 Confocal images

Tissue slices labeled with DAPI & Eosin and with H&E were imaged using a Nikon A1R MP confocal microscope. For DAPI & Eosin staining, excitation was performed at 405 nm for DAPI and 561 nm for Eosin, and signals were collected using DAPI and TRITC filter sets, respectively. For H&E-stained samples, excitation was performed at 488 nm, and fluorescence signal was collected using the FITC filter, in accordance with Soo Heo et al. [48].

### 2.4 Intensity-based imaging

**Fig. 2:**
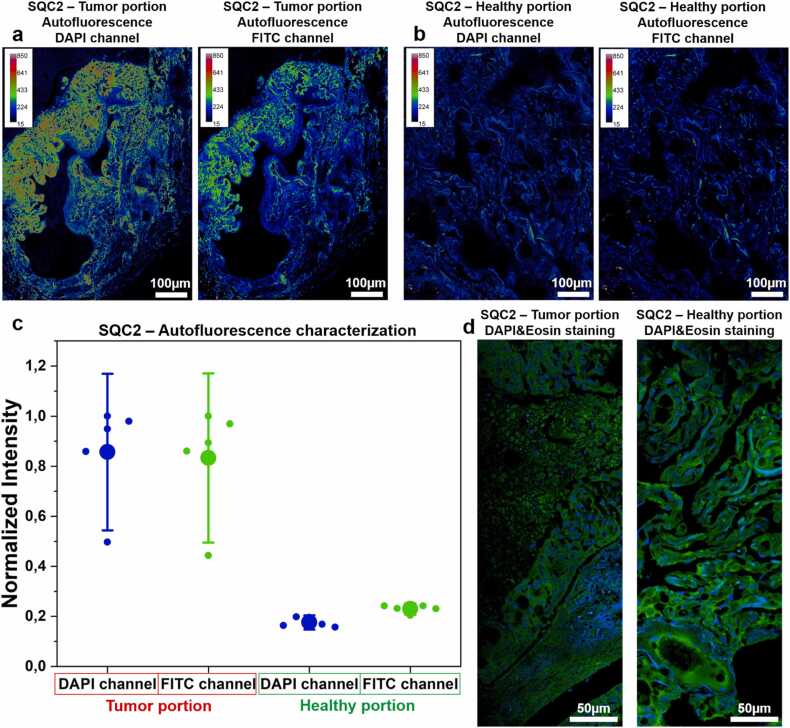
Differences in healthy and cancer portions by exploiting the autofluorescence signal. a) Large image of unstained tumor portion of lung squamous cell carcinoma acquired using an infrared laser (exc. 750 nm). The autofluorescence signal was collected by using DAPI and FITC filters. An RGB Look-Up Table (LUT) was used to map pixel intensity values to corresponding colors. Scale bar 100 µm. Large image’s size of 1.8 × 3.6 mm with an overlap between frames of 20 %. Scale bar 100 µm. b) Large image of unstained healthy portion of lung squamous cell carcinoma acquired using an infrared laser (exc. 750 nm). The autofluorescence signal was collected by using DAPI and FITC filters. An RGB Look-Up Table (LUT) was used to map pixel intensity values to corresponding colors. Scale bar 100 µm. Large image’s size of 1.8 × 3.6 mm with an overlap between frames of 20 %. Scale bar 100 µm. c) Quantification of the autofluorescence signal in both tumor and healthy portion using DAPI & FITC filter, with intensity values normalized to the maximum for each channel. d) Reconstruction of large confocal image mosaics from slices labeled with DAPI and Eosin, using 405 nm and 561 nm excitation wavelengths, respectively. It is important to note that fibers can be excited at 405 nm (due to autofluorescence) and at 561 nm, as a result of eosin staining. Scale bar: 50 µm. The acquisition was performed using NIS software, with an 60x/1.4 objective lens.

For the autofluorescence characterization of 4 μm-thick SQS2 tissue samples mounted in aqueous media shown in Fig. 2, imaging was performed using a Nikon A1R MP system coupled to a multiphoton confocal microscope, with excitation at 750 nm. The signal was collected using the DAPI and FITC channels. An RGB Look-Up Table (LUT) was applied to represent pixel intensity values using Fiji. Each image has a resolution of 1024 × 1024 pixels, with a pixel size of 0.207 µm². For mosaic reconstruction, a 20 % overlap between adjacent images was used. For the autofluorescence analysis in Fig. 2c, the mean intensity values were calculated from 5 different frames, each measuring 156 µm², in both the DAPI and FITC channels.

For the FAD intensity characterization, images acquired using the 810 nm excitation line and collected with a 505/90 nm filter in the healthy region of SQC2 were used. 10 different 10 × 10 µm² frames were selected from both the cellular compartment and fibrous regions.

### 2.5 FLIM experimental setup

The autofluorescence lifetime of various 4 μm-thick tissue samples, mounted in aqueous media, was measured using a Nikon microscope (model Nikon A1R MP system coupled to a multiphoton confocal microscope) equipped with a FastFLIM add-on for fluorescence lifetime imaging (ISS inc., USA). FLIM images were analyzed using the phasor approach. Excitation was performed with a Coherent Chameleon Ultra laser with 80-MHz repetition rate at 750 nm and 810 nm to target NAD(P)H and collagen/elastin, respectively, using a 60X/1.4 NA oil objective. Fluorescence was collected by a large-area PMT after passing through optical filters (450/50 nm for NAD(P)H and 550/90 nm for fiber fluorescence). SHG signal was detected by using 405/10 nm optical filter. FastFLIM computed the fluorescence lifetime for each pixel and directly transferred the data to the phasor plot. The parameters for the lifetime acquisition were fixed: the pixel dwell time was 12 μs, and images were acquired at 512 × 512 pixels with a field of view of 100 μm² (corresponding to a 4 × zoom factor). The excitation power was set to 1.3 (laser power at the sample: 1.1 mW and 7 mW at 750 nm and 810 nm, respectively), with 25 frames accumulated per field of view. Representative intensity-based images from the lifetime acquisitions were obtained by converting the.ifli files to.tiff format using the VistaVision software.

For the characterization of the mounting media (Mount quick aqueous – Bio Optica Milan Spa, Milan, Italy), a small drop (∼250–300 µl) was deposited on a glass, and images were acquired using excitation power set to 4 (laser power at the sample: 10 mW and 9 mW at 750 nm and 810 nm, respectively), with 100 frames accumulated per field of view. We increased both the laser power and the number of frames to enhance the signal and improve photon statistics, due to the intrinsically low fluorescence signal of the mounting. The ISS FastFLIM system was calibrated by measuring mono-exponential lifetime decay of Coumarin in ethanol (Fig. S1) at 750 and 810 nm excitation light, collecting the signal using a 450/50 and 505/90 filter, respectively. To prepare the calibration sample, a stock solution of 100 mmol/L Coumarin in ethanol was prepared and subsequently diluted 1:10 in ethanol. For each measurement, a 512 × 512-pixel FLIM image was acquired until a total of 60 frames were collected.

After mosaic reconstruction (see “Intensity-based imaging section”), we randomly acquired multiple fields of view (FOVs) to ensure representative sampling of the biopsy heterogeneity. For SHG images and their corresponding fluorescence lifetime (SGH + Fluorescence of the same FOV), the following numbers of frames were acquired for the healthy and tumor regions, respectively: 11 and 11 in SQC1; 16 and 16 in SQC2; 14 and 14 in ADC1; 15 and 15 in ADC2; and 18 and 18 in ADC3. For NAD(P)H images, the following number of frames were acquired (and then manually segmented for different analysis) for the healthy and tumor portions, respectively: 20 and 31 in SQC2; 17 and 16 in SQC1; 10 and 10 in ADC2; 10 and 11 in ADC3, and 10 and 11 in ADC1.

### 2.6 Data analysis

The lifetime images were analyzed using VistaVision and SimFCS [35]. Briefly, the intensity decay from each image point is mapped onto the phasor plot as a single point. Specific populations can be selected using a colored cursor, allowing fluorescence intensity images to be color-coded accordingly. This fit-free method enables straightforward FLIM image analysis, as different lifetime populations can be easily isolated and masked.

When a pixel’s intensity decay follows a mono-exponential decay, its corresponding phasor point appears on the universal semicircle. Multi-exponential decays result in points inside the semicircle. The method follows the law of linear combination, meaning that if a pixel contains contributions from multiple exponential components, its phasor position lies along the line connecting those individual components on the semicircle.

**Fig. 3:**
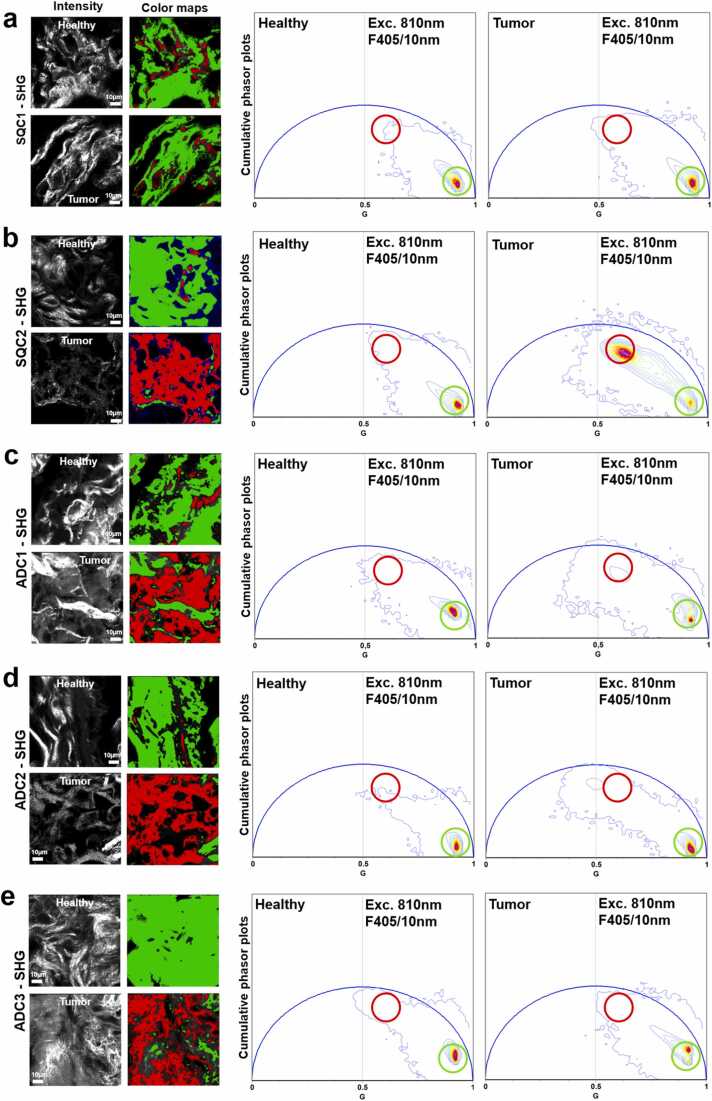
Characteristic SHG phasor plot of health and tumour portion in lung adenocarcinoma and squamous cells. Intensity and colormap of SHG and fluorescence signal collected using the SHG filter (405/10 nm). The fluorescence signal (presumably derived from elastin, red cursor) was acquired using the SHG filter, contributing to the characteristic signature of the tumor portion. Cumulative phasor plot of all frames acquired for each patient of healthy and tumor portions (see material and methods for additional information).

SHG signals, being coherent with the laser, have a lifetime of zero and appear at (s = 0, g = 1) in the phasor plot. In contrast, fluorescence signals from collagen exhibit nonzero lifetimes and appear inside the semicircle. To ensure accurate cursor positioning in the phasor plot, we used VistaVision software. For the analysis of multiple images, we employed SimFCS. As shown in Fig. 3, the cursor was manually placed using SimFCS to highlight the long-lifetime species. Finally, intensity and area analyses were performed using custom-made macros in Fiji, with results plotted in Origin2024.

The Elastin/Collagen Index (ECI) is a normalized measure used to assess the balance between elastin and collagen in each tissue. ECI is commonly used to investigate the roles of elastin and collagen in human dermal aging [49], [50], by quantifying their amounts as a function of dermal depth and age. For each 100 µm² FOV, we quantified the area of elastin (excitation at 810 nm, filter 505/90) and collagen (excitation at 810 nm, filter 405/10) in the same FOV and subsequently calculated the ECI index. Fiber areas were measured after applying a dedicated thresholding routine that efficiently segmented fibers from the cellular component. ECI is calculated using the formula:

$$\mathrm{ECI}=\frac{\left(\mathrm{Elastin Area}-\mathrm{Collagen Area}\right)}{\left(\mathrm{Elastin Area}+\mathrm{Collagen Area}\right)}$$

The numerator (Elastin - Collagen) represents the difference between the two components, indicating changes in the Elastin/Collagen ratio. The denominator (Elastin + Collagen) normalizes the result, ensuring that the index remains within the range of −1 to + 1.

For elastin quantification in H&E-stained tissue slices, 6 different regions of interest (ROIs) of 65 × 65 µm² were selected and analyzed using Fiji. Signal quantification was performed using the integrated density, defined as the product of the area and the mean gray value.

## 3 Results

### 3.1 Autofluorescence signatures in healthy and tumor tissues in lung

Fig. 1a illustrates the experimental workflow used for analyzing lung adenocarcinoma and squamous cell carcinoma. First, the human derived lung cancer biopsies were extracted and fixed in formalin and embedded in paraffin for preserving the biological features. Then, the samples were cut in thin slices of ∼4 µm, deparaffinized and preserved in an aqueous mounting media for sample preservation. Unstained biopsies were imaged using an infrared laser, and the optical signatures of healthy and tumor regions were examined in different emission channels through phasor-plot analysis (Fig. 1b). At this stage, a rapid assessment of the biological characteristics can be performed by analyzing the differential expression of various molecules within the healthy and tumor regions of LC without the need for specific staining. Validation was performed using standard H&E staining (Fig. 1c – f). The histological features of poorly differentiated squamous cell carcinoma (SQC1) and adenocarcinoma (ADC1) are presented. Fig. 1c and d show squamous cell carcinoma characterized by an infiltrative growth pattern with irregular tumor nests interspersed with fibrotic tissue. Fig. 1e and f display adenocarcinoma with solid tumor sheets and micropapillary structures surrounded by fibrous stroma.

The endogenous fluorescence of cells and fibers was well preserved in formalin-fixed, deparaffinized, and unstained tissue sections in both SQC and ADC. The fixation process stabilizes tissue architecture while modifying protein conformation [51]. Formaldehyde reacts with free amino groups in nucleotides and proteins, forming hydroxymethyl compounds that facilitate crosslinking via methylene bridges [52], [53]. For this reason, when describing a specific biomarker under investigation, we usually refer to it as a mixture of biomarkers and formalin. In addition to chemical fixation, we tested the fluorescence properties of the mounting medium. We observed that the aqueous mounting medium used to preserve formalin-fixed slices exhibits low autofluorescence and a longer lifetime at 750 nm and 810 nm excitation wavelengths compared to the samples and does not interfere with our measurements (Fig. S2).

Fig. 2a and b show representative large-scale images of healthy and tumor tissues of SQC2 sample. To validate the structural preservation of the specimens, the samples were excited at 750 nm, and the resulting intensity signal was collected using the DAPI and FITC channels to obtain a detailed map of the sample features in both the tumor (Fig. 2a) and healthy (Fig. 2b) region. Mosaic reconstruction was performed to analyze a large FOV (Fig. 2a and b). Interestingly, the tumor regions exhibited higher autofluorescence compared to healthy areas in both DAPI and FITC channels, likely due to a denser deposition of fibrous material (Fig. 2c). Notably, differences in the distribution and density of cellular and fibrous components highlight distinct architectural features between healthy and tumor tissues. The tumor portion of SQC2 demonstrated pronounced fibril deposition, whereas the healthy tissue exhibited a balanced distribution of cells and fibrils. These differences were also demonstrated using a confocal microscope by exploiting the DAPI & Eosin [1] costaining, to label nuclei and basic cellular compartments (Fig. 2d), and it was consistently observed across nearly all samples investigated (see images of other samples at different zoom in Fig. S3).

Based on the two-photon absorption cross-section [11] and as noted in previous multiphoton fluorescence studies [54], [55], the fluorescence observed at 750 nm excitation originated from NAD(P)H, a metabolite known for its autofluorescent properties while the autofluorescence of fibrillar structure can be attributed to collagen in which hydroxylysyl pyridinoline and lysyl pyridinoline crosslinkers act as fluorescent structure. Interestingly, the autofluorescence of collagen enabled detailed analysis of tissue architecture and the spatial relationship between cells and the extracellular matrix (ECM), even in these unstained slices.

### 3.2 Phasor-FLIM reveals ECM remodeling in lung tumors via SHG and autofluorescence

Tissue autofluorescence enables the characterization of the cytoarchitecture in biopsies. Based on the autofluorescence characterization and the morphological features shown in Fig. 2, we observed that lung tumor samples often exhibit a high degree of fiber deposition. This process, known as fibrosis, involves excessive fiber accumulation, leading to structural changes and dysfunctional lung function.

We investigated the distribution and spectral characteristics of these fibers using FLIM-phasor approach (Fig. 3a – e). Using an excitation wavelength of 810 nm, we collected signals at 405/10 nm (SHG). As described by Gratton et al. [36], the relative intensity of SHG signal is highly dependent on collagen types. Specifically, collagens I and II produce strong SHG signals, while collagen III contributes weakly [36].

By analyzing the SHG signal in both healthy and tumor samples (Ex. 810 nm; filter 405/10), the phasor plot revealed significant differences in almost all patients investigated. In healthy samples, excitation at 810 nm typically produced a strong SHG signal, with all pixels in the frame localized at coordinates s = 0 and g = 1. Green-colored cursor in Fig. 3b – e exhibits the major clusters in the phasor distribution originating from the fibers of the healthy sample portion, with the corresponding image color-coded accordingly. In contrast, tumor specimens exhibit a distinct phasor plot distribution, where the red-colored cursor, positioned at a longer lifetime, selects the distribution originating from other fibers of tumor specimens (Fig. 3b – e).

Specifically, the weak SHG contribution in tumor samples was largely obscured by the leakage of a very strong fluorescence signal collected by the filters used to separate SHG signals. This effect can be attributed to elastin, which exhibits the most intense fluorescence among the fibers investigated. Biopsies from four different patients consistently displayed a similar phasor plot when using the SHG filter, indicating a mixture of the SHG signal – primarily associated with healthy tissue – and fluorescence emission originating from tumor specimens. The cumulative phasor plot, generated from SHG frames, reveals variations in the SHG signal. SQC2 exhibits the most pronounced difference between healthy and diseased regions, with fibers characterized by a longer lifetime (Fig. 3b; see red cursor). Similarly, in ADC1 (Fig. 3c), ADC2 (Fig. 3d), and ADC3 (Fig. 3e), we observed alterations in fiber characteristics, indicating modifications in the ECM. However, one patient (SQC1, Fig. 3a) did not exhibit significant differences between healthy and diseased tissues, showing no clear phasor plot alteration. This lack of response suggests possible variability in disease progression or individual differences in tissue composition.

### 3.3 Phasor-based identification of fiber remodeling in lung cancer

**Fig. 4:**
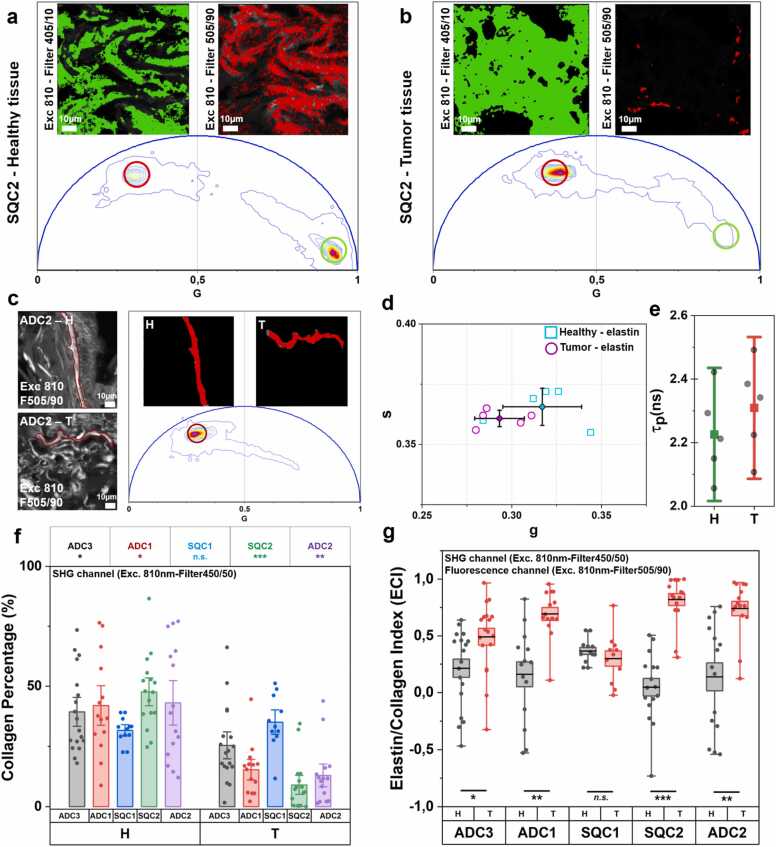
Fiber characterization by using the optical signature of tissue and differences in collagen percentage and averaged ECI: Phasor-plot optical signature of fluorescence (long lifetime) and SHG signal (short lifetime) in healthy a) and tumor b) portion of SQC2. c) Manual fiber segmentation and optical signature in the phasor plot in both heathy (in green) and tumor (in red) portions, with the corresponding phasor-plot coordinate d) and average phase lifetime e). f) Collagen percentage in ADC and SQC in healthy and tumor portion. g) ECI showing an unbalanced ratio between elastin and collagen in tumor portions respect to healthy regions, except for SQC1. H and T refer to the healthy and tumor portion, respectively. Error bars show mean ± SE. A non-parametric Wilcoxon signed-rank test was performed (n.s. – not significant, *P < 0.05, **P < 0.01, ***P < 0.001).

We hypothesized that tumor samples exhibit elevated elastin accumulation, a phenomenon known as elastosis. Using an excitation wavelength of 810 nm, we collect emission signals at 505/90 nm and 405/10 nm to investigate the phasor characteristics of the fiber in the fluorescence domain, extending the analysis beyond the SHG signal. In the phasor plot, SHG signals appear at coordinates s = 0 and g = 1, reflecting their zero lifetime, while fluorescence signals are associated with long-lifetime characteristic (Fig. 4a). Collagen is expected to produce both fluorescence and SHG signals; however, the minimal overlap between these channels suggests the presence of two distinct fiber populations (Fig. 4a and b).

To determine the nature of the fluorescent fibers at 505/90 nm and identify their type, we analyzed the average phase lifetime and the emission spectrum in both healthy and tumor specimens. Various images were manually segmented to extract information on the phasor coordinates and average fluorescence lifetime in both healthy and tumor regions (Fig. 4c and d and for other samples, see Fig. S4). The average phase lifetimes are shown in Fig. 4e for ADC2. Compared to collagen, which has a mean phase lifetime of 1.5 ns [36], the acquired fiber exhibited an average phase lifetime of approximately 2.2 ns, indicating a longer phase lifetime than collagen (Fig. 4e). Moreover, both healthy and tumor samples showed similar values, suggesting comparable spectral characteristics of the fluorescence signal (healthy: 2.23 ± 0.14 ns; tumor: 2.31 ± 0.15 ns) (Fig. 4e). This suggest the presence of an additional fiber type, which can be characterized by using phasor-FLIM analysis. Then, the fluorescence spectrum of fibrous patterns was obtained to understand their absorption characteristics (Fig. S5). In agreement with Li et al. [16], we observed that the curved fibers in both healthy and tumor samples exhibited an emission spectrum characteristic of elastin, with a peak at the 484 nm. Furthermore, consistent with Tilbury et al. [56], we confirmed that the SHG signal originates from collagen, while the fluorescence signal is associated with elastin in lung sample.

Interestingly, in healthy lung tissue, we observed a balance between elastin (red, fluorescence signal) and collagen (green, SHG signal). In normal tissues, elastin was organized within the structural framework of collagen, reflecting a well-structured fiber network. However, in diseased tissue, elastin appeared disorganized and was not mixed with collagen fibers (Fig. S6). Elastic fiber formation also increases in other lung pathologies, such as idiopathic fibrosis [56], where the elastin/collagen ratio may be altered during disease progression.

To investigate these changes in lung cancer, we analyzed both collagen (SHG) and elastin (TPEF) in the same FOV of healthy and tumor-affected tissues for each patient. The autofluorescence intensity from cellular components at the excitation wavelength of 810 nm (i.e., FAD) was significantly weaker than that of elastin, allowing for successful fiber segmentation via thresholding (Fig. S7). Similarly, in SHG images, the strong signal enabled efficient segmentation using customized thresholding.

Fig. S8 present a representative thresholded and segmented images of elastin in normal and tumor tissues. As shown in Fig. S8, the segmentation of images by thresholding shows a similarity to the color-map obtained by positioning the cursor in the phasor plot to identify a population with a specific lifetime. By quantifying the segmented area, we determined the percentage of collagen in both healthy and tumor samples. In Fig. 4f, the SHG area was significantly reduced in the tumor portion, indicating a corresponding decrease in collagen content. Moreover, a comparison of SHG signals between healthy and tumor regions in SQC2, ADC1, ADC2, and ADC3 revealed a marked reduction in collagen per frame in tumor samples.

Finally, the ECI, calculated from all parenchymal imaging in both normal and tumor regions, is presented in Fig. 4g. The numerator (Elastin - Collagen) of the equation represents the difference between the two components, indicating an unbalanced in the tissue. Meanwhile, the denominator (Elastin + Collagen) acts as a normalization factor, ensuring that the index remains within a standardized range of −1 to + 1 (see in the Methods section). The resulting values reveal a more balanced elastin-to-collagen ratio in normal lung tissue (ECI close to 0), whereas the tumor region exhibits a significantly higher index, approaching 1. In this observation, SQC1 does not show any variation.

### 3.4 Complementary fluorescent staining reveals tumor-associated elastin deposition

To validate the FLIM observations, we complemented the label-free characterization with a fluorescent staining approach. As demonstrated by Soo Heo et al. [48], elastic fibers can be visualized using conventional H&E staining. Indeed, the hematoxylin staining quenches the excessive eosin fluorescence emitted by other tissue components, thereby enhancing the contrast of elastic fibers.

**Fig. 5:**
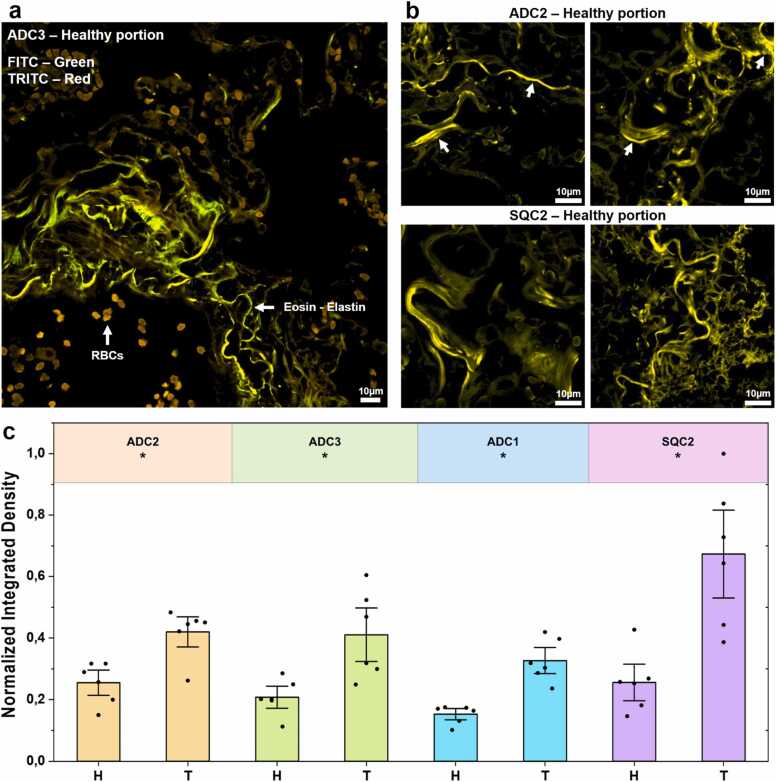
H&E staining for the validation of elastin deposition. (a) Confocal image of a large vessel in the healthy region of ADC3. In the FITC channel, a strong signal from elastic fibers (white arrows) is observed, while autofluorescence from red blood cells (RBCs) appears in the TRITC channel. Confocal images; excitation at 488 and 561 nm; scale bar: 10 µm. (b) Comparison between healthy and tumor regions of H&E-stained tissue slices acquired using a confocal microscope. Elastic fibers were detected at 488 nm, revealing a marked accumulation of elastin in the tumor region. Confocal image; excitation at 488 nm; scale bar: 10 µm. (c) Quantification of labeled elastin with statistical analysis using the non-parametric Wilcoxon signed-rank test (*P < 0.05). Values were normalized to the maximum value across all samples. Error bars show mean ± SE. H and T refer to the healthy and tumor portions, respectively.

Following staining, we analyzed the tissue slices using a fluorescence microscope. As shown in Fig. 5a, when imaging a vessel in the healthy lung region, elastic fibers were clearly distinguishable due to their strong fluorescence signal, in contrast to the surrounding biological structures. Notably, the curved, fibrous architecture of elastin within the arterial wall closely resembled the pattern observed in both optical [57] and fluorescence images [16]. This confirms that eosin – in tissue slices labelled for H&E – enhances elastin contrast under fluorescence microscopy in lung tissue.

We then compared healthy and tumor regions in samples that previously exhibited differences in label-free imaging. As illustrated in Fig. 5b, a marked increase in elastin deposition was observed in the tumor areas. Furthermore, we quantified the fluorescence signal attributed to elastin and found a trend consistent with the label-free FLIM results (Fig. 5c). Specifically, in four patient samples, we confirmed a pronounced accumulation of elastin within the tumor region, supporting the findings obtained through label-free analysis.

### 3.5 Comprehensive tissue characterization using phasor-FLIM

**Fig. 6:**
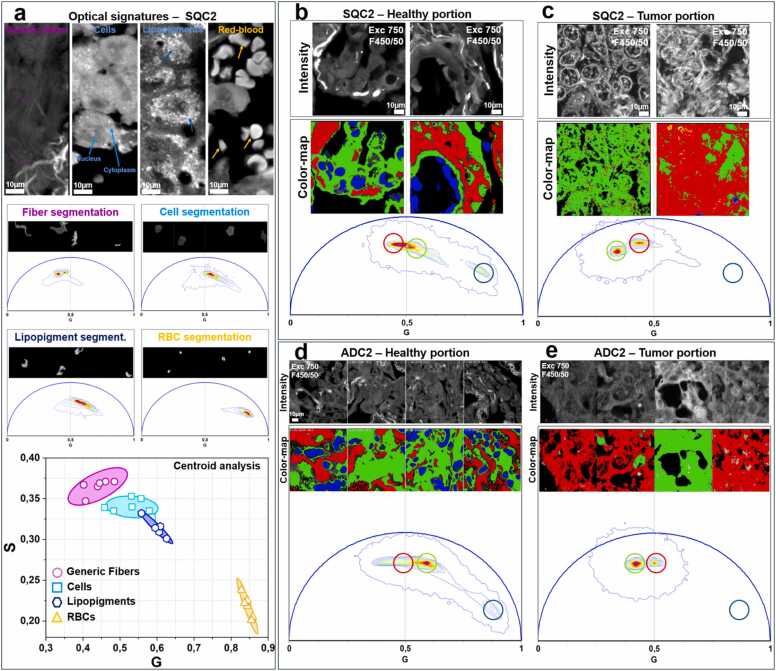
(a) Characterization of tissue components in the healthy region of SQC2. Autofluorescence images of fibers, cells, lipopigments, and red blood cells (RBCs) are shown at the top (the regions of interest are highlighted by the arrows). Manual segmentation of the different components and their corresponding optical fingerprints are shown in the middle panel. Centroid analysis of the segmented components in the phasor plot is presented at the bottom. (b-e) Optical signatures of healthy tissue and tumor portion of SQC2 and ADC2: Phasor-FLIM allows the separation of cells (in green in the color-map), fibers (in red in the color-map) and red blood cells (in blue in the color-map) in heathy (b) and tumor (c) portion of SQC2. Scale bar 10 µm. Phasor-FLIM allows the separation of cells (in green in the color-map), fibers (in red in the color-map) and red blood cells (in blue in the color-map) in heathy (d) and tumor (e) portion of ADC2. Scale bar 10 µm. Exc. 750 nm, filter 450/50.

Beyond fiber-derived signals, phasor-FLIM analysis was employed to explore additional optical signatures in both healthy and tumor regions. We used the healthy portion of the SQC2 sample as a reference to characterize the optical signatures of different biological components. To achieve this, we manually segmented various cellular and non-cellular features to obtain accurate fluorescence lifetime values. Based on intensity images acquired at 750 nm excitation, we could distinguish different tissue components – such as fibers, cells, lipopigments, and red blood cells (Fig. 6a, top). Each biological feature was then manually segmented to determine its corresponding position in the phasor plot (Fig. 6a, middle). Finally, we calculated the centroid of each segmented region to precisely determine the optical signature of each component. As shown in Fig. 6a (bottom), fibers exhibit longer lifetimes compared to cells; lipopigments show lower lifetime respect to cells, whereas red blood cells (RBCs) are characterized by short lifetimes. Then, the phasor plot was used to characterize the differences between healthy and tumor regions. The phasor approach enabled clear discrimination between healthy and tumor tissues based on their distinct fluorescence lifetime characteristics (Fig. 6b – e). In sample SQC2, tumor regions exhibited longer fluorescence lifetimes in cellular components compared to their healthy counterparts, as visualized in the green color map in Fig. 6b and c. This trend was consistent across all analyzed samples, including both ADC and SQC subtypes (Figs. 6d, e and S9).

Two additional fingerprints were characterized in healthy slides. Some cells in healthy tissue sections exhibited lipofuscin-like accumulations (or ceroid), characterized by short fluorescence lifetimes, consistent with recent findings [28] (Fig. S10). As shown in the blue color map in Figs. 6 and S9, an additional key optical fingerprint of healthy tissue was the prominent presence of red blood cells (RBCs) exhibiting short fluorescence lifetimes, which was absent in tumor samples. It is well established that RBC autofluorescence under two-photon excitation in the 700–800 nm range originates from hemoglobin [58], [59]. All these molecular markers in the phasor plot allow differentiation between the healthy portion and the tumor part in unstained sample slides.

## 4 Conclusion

In this work, we analyzed two different histotypes of NSCLC using the phasor-FLIM approach. Combined with traditional pathology, FLIM can provide complementary information from unlabeled tissue slices. Although structural changes in cancer progression can be observed by trained pathologists using specific contrast agents (e.g., nuclear aberrations with H&E staining), FLIM extracts additional quantitative data from unstained tissue samples, enhancing pathologists’ understanding and improving tumor diagnosis. Our results suggest that performing phasor-FLIM could complement traditional histopathology, allowing for advanced disease assessment by integrating label-free fluorescence imaging with contrast-based diagnosis. In the clinical context, the label-free characterization process, which includes the extraction and fixation of human lung cancer biopsies to the label-free characterization, takes approximately 2–3 days, thereby reducing the time required for more specific and molecular-level diagnoses. Subsequently, the diagnosis is confirmed through the gold-standard H&E staining, evaluated by trained pathologists. Regarding biochemical changes in cells and tissues, FLIM shows great potential for cancer detection. This work lays the foundation for developing minimally invasive optical screening techniques and may complement existing methods by enhancing sensitivity in the clinical context.

Several key molecules can be exploited in the FLIM approach. First, NAD(P)H and FAD are among the most studied molecules in biological systems [28], [29], [31], [33], [34], [35], [52]. These endogenous coenzymes are involved in different cellular metabolic reactions such as glycolysis and oxidative phosphorylation. NAD(P)H and FAD exist in two states: free or protein-bound, each exhibiting distinct fluorescence lifetimes. Free NAD(P)H is characterized by a short lifetime, whereas its protein-bound form has a longer lifetime [30], [32]. In contrast, free FAD displays a long lifetime, while the protein-bound form shows a shorter lifetime [60]. The balance between bound and free states of these molecules described are highly sensitive parameters for understanding cellular metabolism. In most tumor types, cancer cells exhibit an altered metabolism, a phenomenon known as the Warburg effect [61], [62], [63]. In this process, tumor cells preferentially rely on oxygen-independent glycolysis rather than mitochondrial oxidative phosphorylation, even when oxygen is available. However, recent models suggest that cancer-associated fibroblasts (CAFs) play a crucial role in tumor metabolism through a mechanism known as the “reverse Warburg effect” [64], [65]. From a metabolic perspective, FLIM can serve as a crucial tool in defining the role of this intricate network. Key studies on cellular metabolism using FLIM have primarily been conducted on cell cultures, where cancer cells and their healthy counterparts were analyzed separately. FLIM has been successfully employed to detect metabolic changes in non-tumorigenic cells (MCF10A), non-invasive cancer cells (MCF-7), and invasive cancer cells (MDA-MB-231), based on variations in fluorescence lifetime [66], [67]. In addition to lifetime-based approaches, other methods exploiting endogenous autofluorescence rely on fluorescence intensity have been used for metabolism understanding. In particular, the optical redox ratio – defined as the ratio of NAD(P)H to FAD fluorescence intensity – provides a quantitative measure of the relative oxidation-reduction state of individual cells [68], [69]. This metric has been applied to investigate the metabolic state of various cell types, including tumor-associated populations such as cancer cells [70], [71] and immune cells [72]. Although these studies used living cells, the use of specific cell types reduces biological complexity and may lead to oversimplified or potentially misleading interpretations. Although challenging, exploiting tissue autofluorescence represents a powerful approach to investigate tumor characteristics and fibrosis. Several studies have employed FLIM to analyze cancer biopsies and pathogen-induced fibrosis, without using phasor analysis and focusing only on a limited set of biological features [41], [73], [74]. In our study, we investigated LC by distinguishing different endogenous components within the tissue. This was achieved through the segmentation of distinct molecules of interest combined with phasor analysis, which enable a more refined characterization of the sample’s optical signatures. In this context, our approach allowed high spatial resolution mapping of various tissue components, assigning specific optical fingerprints to each biological species and deciphering changes occurring within the tumor regions. This approach represents a valuable tool for investigating the molecular characteristics of biopsies and for generating more detailed medical reports. Interestingly, several studies have demonstrated the preservation of metabolic cofactors in fixed tissues and FFPE tissue sections [40], [41], [75], [76]. However, Sànchez-Hernàndez et al. [53] reported difficulties in attributing NAD(P)H and FAD signals in formalin-fixed slides. The fixation process alters the fluorescence spectrum and lifetime, particularly in the NAD(P)H emission range. Hence, identifying the primary contributing metabolic fluorophores in fixed specimens is challenging and depends on factors such as sample type, fixation duration, and the specific chemical fixation method used. Future research should aim to address these limitations by investigating the metabolic activity in cancer and their interaction with the ECM, using living biopsies.

In our study, by setting our measurements to detect fluorescence of NAD(P)H, we observed variations in the lifetime across different cellular compartments. An important characteristic of tumor regions, as highlighted by phasor-FLIM, is the reduction of RBCs, indicating a highly anoxic state within the tumor. This is particularly evident in the tumor portions, which show high fiber production, which contributed to the creation of anoxic environments. Also, we observed longer fluorescence lifetimes in tumor regions compared to healthy tissue in both ADC and SQC using 750 nm excitation wavelength and NAD(P)H filter. Although we cannot exclude the contribution of formalin to preserve the biological specimens, we hypothesize that metabolic differences exist between healthy and tumor regions at the cellular level. In contrast to previous studies on cell models, where cancer cells typically show shorter fluorescence lifetimes due to a predominantly glycolytic metabolism (the so-called Warburg effect) [39], [75], our findings reveal the opposite trend: the cancer cells exhibit a longer lifetime signature, indicative of a more oxidative metabolic profile. Reactive oxygen species (ROS) may help explain why tumor tissues display longer fluorescence lifetimes. Gratton and his team found that FLIM can detect ROS production and lipid peroxidation [77], which create a distinct, long-lifetime signature in the NAD(P)H channel. Cancer cells produce more ROS than healthy cells because their metabolism shifts and their mitochondria become less efficient. Indeed, the ROS formation actively shape the disease by triggering tumor formation, fueling rapid growth, enabling cancer cells to invade surrounding tissues and spread, and even helping tumors resist chemotherapy [78]. When FLIM detects long lifetime species in tumor regions, it is likely reflecting the high levels of ROS in those areas. Additionally, changes in the local microenvironment – such as shifts in pH, variations in oxygen concentration, and differences in refractive index – can further modulate fluorescence lifetime by affecting non‑radiative decay pathways and fluorophore interactions [41]. A shift toward longer lifetime in tumour cells suggests microenvironmental differences in the tumour regions, which include, for example, altered expression and protein binding, viscosity, or refractive index.

Concerning the other enzymatic cofactor, FAD is not excitable at 750 nm [79], so we used 810 nm excitation. However, in all analyzed lung samples, the autofluorescence signal from tissue fibers was significantly stronger than that of the enzymatic cofactor.

Additional autofluorescent molecules include elastin and collagen bundles. Under physiological conditions, collagen plays a crucial role in regulating the wound-healing process, including cell differentiation, proliferation, adhesion, and migration, while also maintaining tissue architecture and organization [80]. However, in cancer, these processes can become aberrantly activated, leading to the development of desmoplasia, a fibrotic deposition within the tumor microenvironment [45]. In the lungs, elastin and collagen are the primary components of the connective tissue network, working together to ensure elasticity and tensile strength [57]. Numerous studies have demonstrated that FLIM and SHG microscopy are effective methods for distinguishing collagen from elastin [16], [56], [73], [81]. In our measurements, we discriminated collagen fibers from elastin bundles using two-photon excitation at 810 nm, collecting the SHG signal at 405/10 nm and fluorescence at 505/90 nm, respectively. Phasor-FLIM analysis indicates that normal tissue maintains a balance between elastin and collagen, whereas in tumor conditions, aberrant elastin secretion is often observed. This excessive elastin production, known as elastosis [82], is frequently overlooked in favor of collagen, due to its more apparent role in fibrosis in many fibroproliferative diseases. However, recent findings highlight elastin's significance. Growing evidence suggests that excessive elastin deposition occurs in both acute and progressive airway and parenchymal pulmonary disorders, significantly contributing to disease progression, morbidity, and mortality. In agreement with this, Fukushima et al. [83] demonstrated, using light microscopy, electron microscopy, and immunocytochemistry, that elastosis was detected in 80.2 % of ADC, 18.3 % of SQC, and 71.4 % of adenosquamous carcinomas. These results are consistent with our data: out of five patients with ADC and SQC, elastosis was detected in four samples from unstained tissue sections using the phasor-FLIM approach. All ADC samples showed a high elastin content in the ECM, while only one SQC biopsy exhibited elastosis, supporting a correlation with the cited study. This suggests that elastosis may play a role in tumorigenesis in NSCLC, particularly in ADC compared to other histotypes. However, the specific contribution of elastosis to disease progression remains unclear. Recent studies have shown that adenocarcinomas with elastosis exhibit significantly improved survival rates compared to those without [83]. Additionally, a low degree of collagenization or hyalinization in the fibrotic focus at the tumor’s center has been correlated with better prognosis [84], [85]. Indeed, high elastin expression in the tumor matrix was significantly associated with smaller tumors, measuring less than 3 cm [86]. Similarly, adenocarcinomas that grow by replacing alveolar lining cells are associated with improved outcomes compared to those exhibiting expansive and destructive growth [87].

Although this method holds potential for clinical application, the process requires further automation to be practical in routine diagnostics. In the future, machine learning (ML) will be employed to enhance and automate phasor analysis in FLIM, particularly for the identification and quantification of endogenous fluorophore populations in complex biological samples. Rather than relying on manual region selection within phasor plots, ML algorithms can be trained to automatically classify and analyze the data, thereby improving both efficiency and accuracy. Overall, the information obtained through FLIM and SHG can help elucidate the mechanisms of tumorigenesis in NSCLC, broadening their applicability in both medical and basic research.

## CRediT authorship contribution statement

**Alberto Diaspro:** Writing – review & editing. **Marco Lucchi:** Writing – review & editing. **Mastromarino Maria:** Writing – review & editing, Methodology. **Greta Alì:** Writing – review & editing, Investigation. **Nicola Belcari:** Writing – review & editing, Funding acquisition. **Luca Pesce:** Writing – review & editing, Writing – original draft, Software, Resources, Methodology, Investigation, Formal analysis, Data curation, Conceptualization. **Marco Scotto:** Methodology. **Giuseppe Vicidomini:** Methodology. **Cristina Niccoli:** Methodology. **Giuseppe Sancataldo:** Writing – review & editing. **Paolo Bianchini:** Investigation.
