# Supplementary material for: Phasor-FLIM and SHG imaging for quantitative analysis of lung cancer autofluorescence
Source: Comput Struct Biotechnol J. 2025 Aug 12;30:80–93. doi: 10.1016/j.csbj.2025.08.010 (PMC12392685; doi:10.1016/j.csbj.2025.08.010)
Supplement: Supplementary file 1 — Supplementary material [file mmc1.docx]

**Supplementary information**

Figure S1. System calibration at two different excitation wavelength (750 and 810nm) using a solution of Coumarin in ethanol.


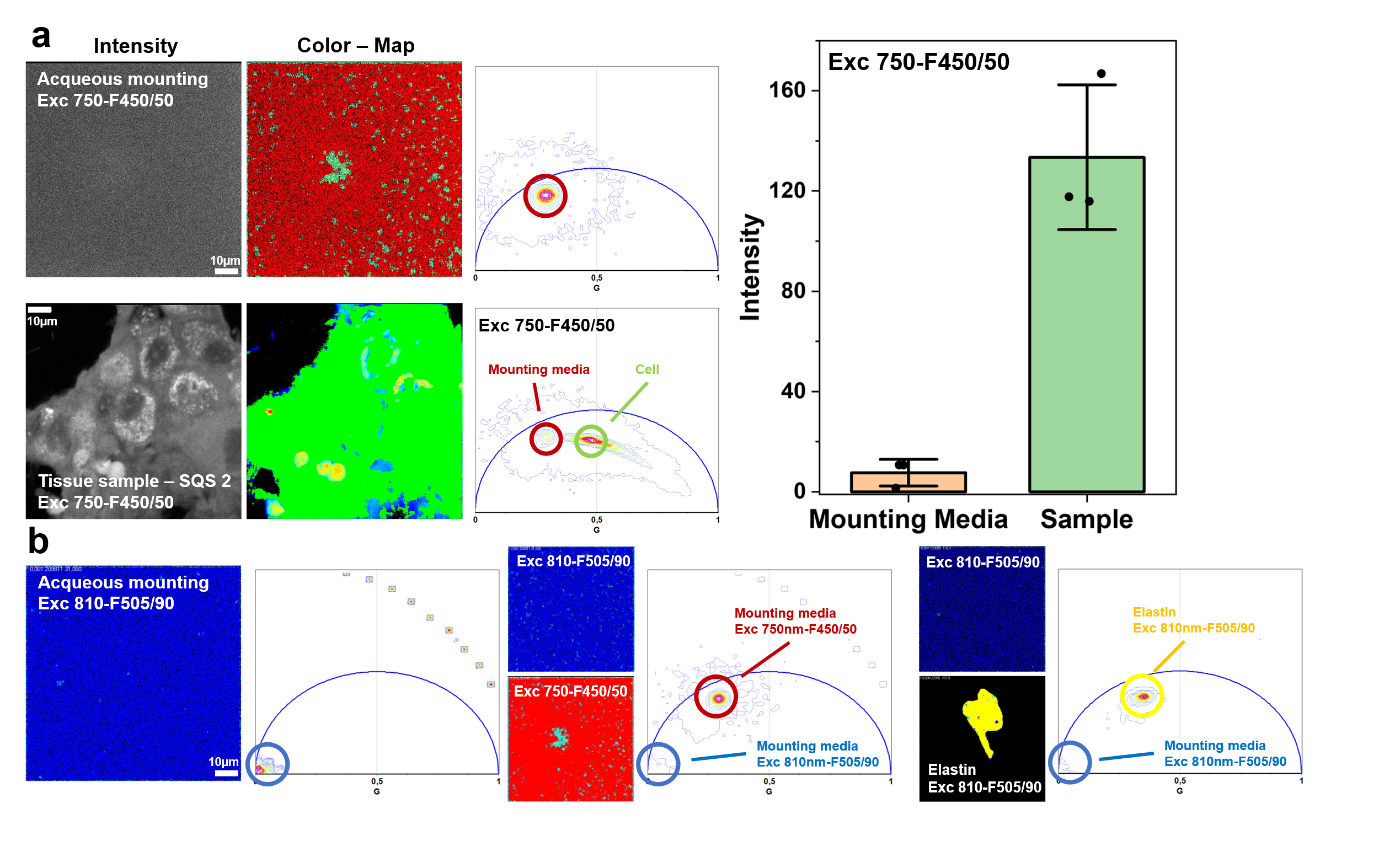


Figure S2. a) Characterization of the aqueous mounting medium at 750 nm excitation wavelength compared to biological tissue, using the phasor plot method and fluorescence intensity. The comparison was performed with different laser powers (tissue: 1.1 mW; mounting medium: 10 mW, as now reported in the Methods section) and frame accumulation settings (25 frames for tissue; 100 frames for mounting medium). Scale bar: 10 µm. b) Characterization of the aqueous mounting medium at 810 nm excitation wavelength. The observed long-lifetime signature and low-intensity signal confirm the low excitability of the mounting medium at 810 nm. The panel also includes a comparison between the mounting medium at 750 nm and 810 nm excitation wavelengths, as well as a comparison between the mounting medium and segmented elastic fibers at 810 nm excitation.


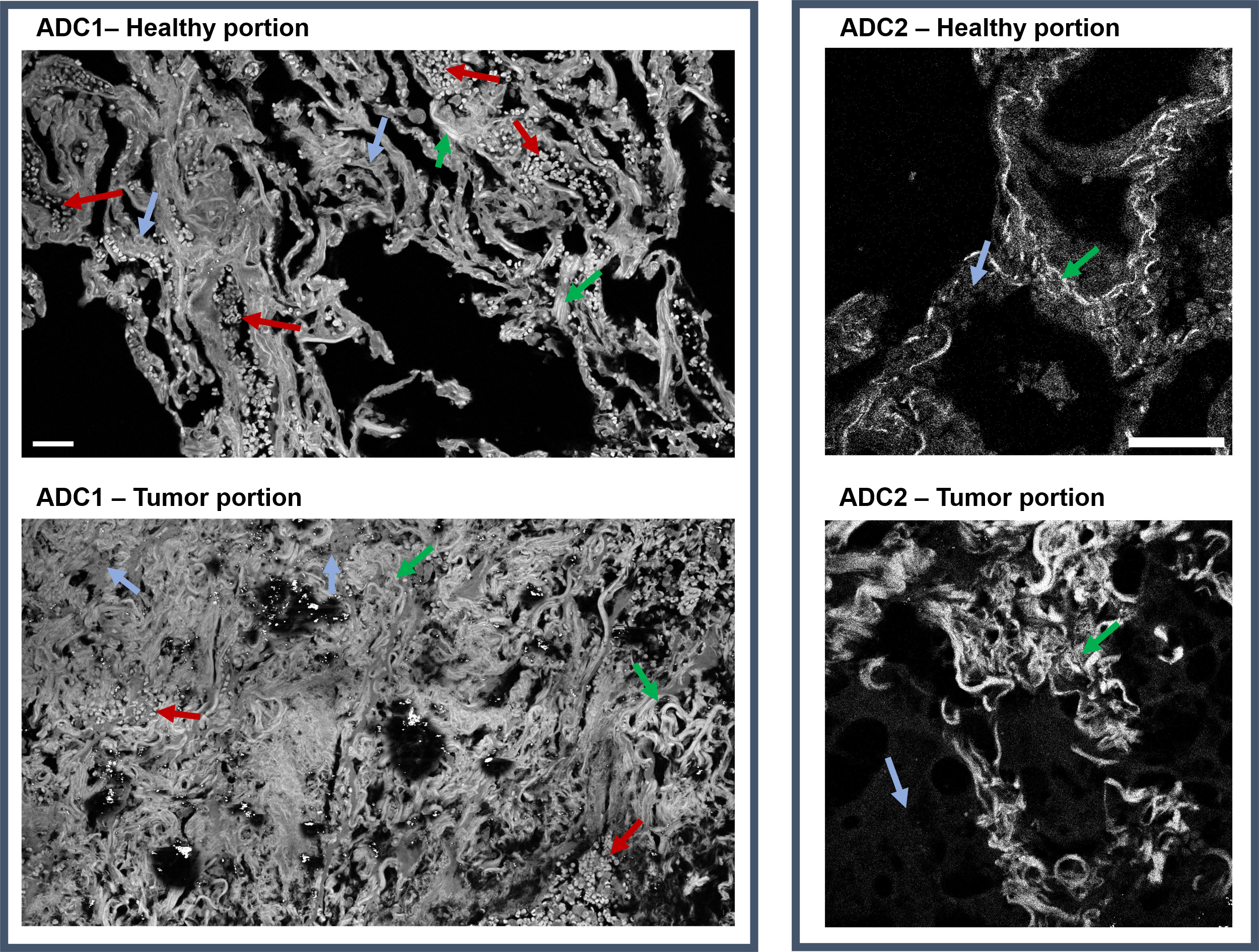


Figure S3. Two-photon imaging of healthy and tumor portion of the samples ADC1 and ADC2. Blue arrow: cellular compartments; red arrow: red blood cells; green arrow: fibers. Scale bar 200 µm for ADC1 and 50 µm for ADC2.


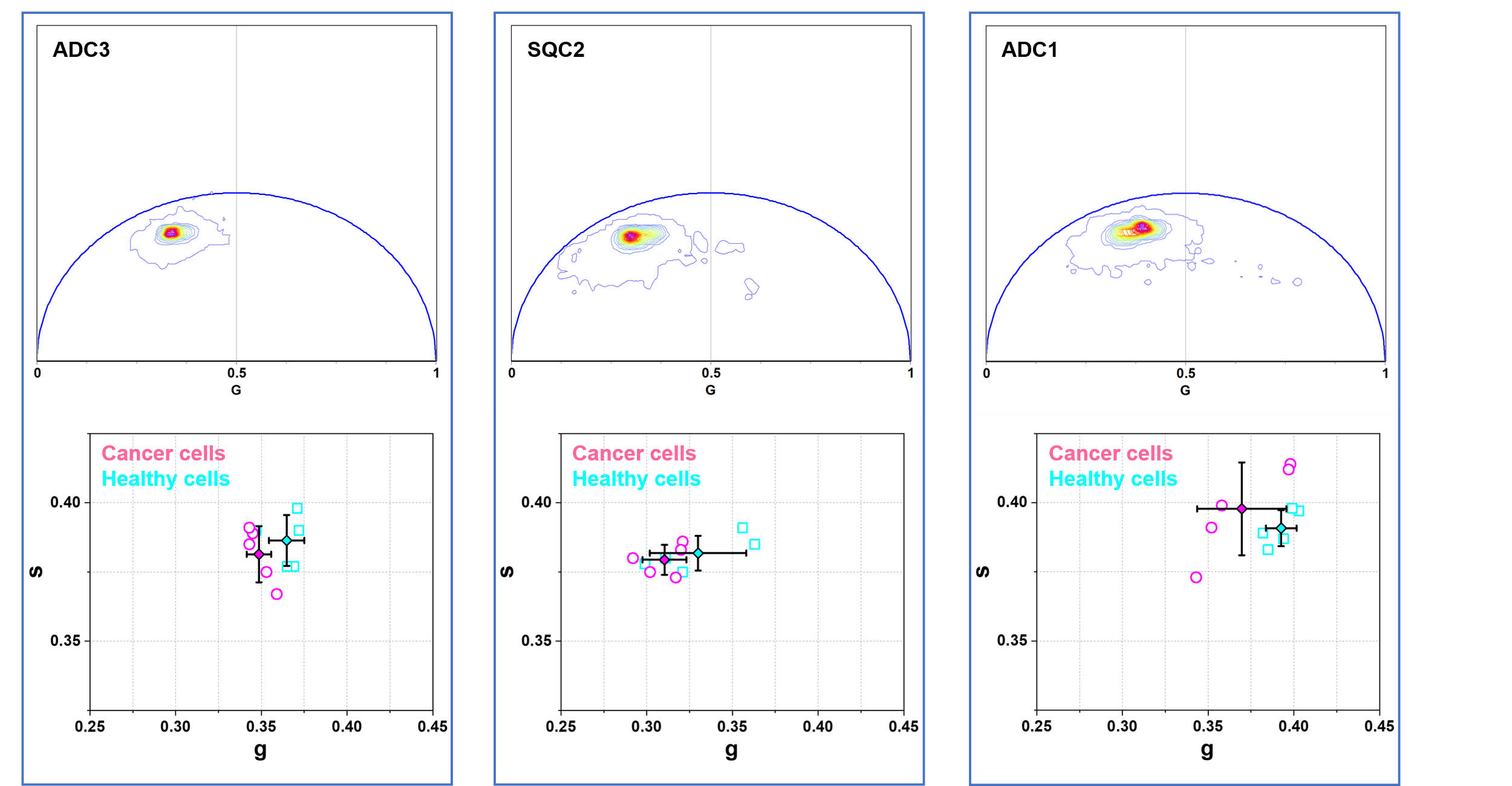


Figure S4. Fiber segmentation in healthy and tumor regions of ADC3, SQC2 and ADC1. Excitation light 810nm, filter 505/90nm.


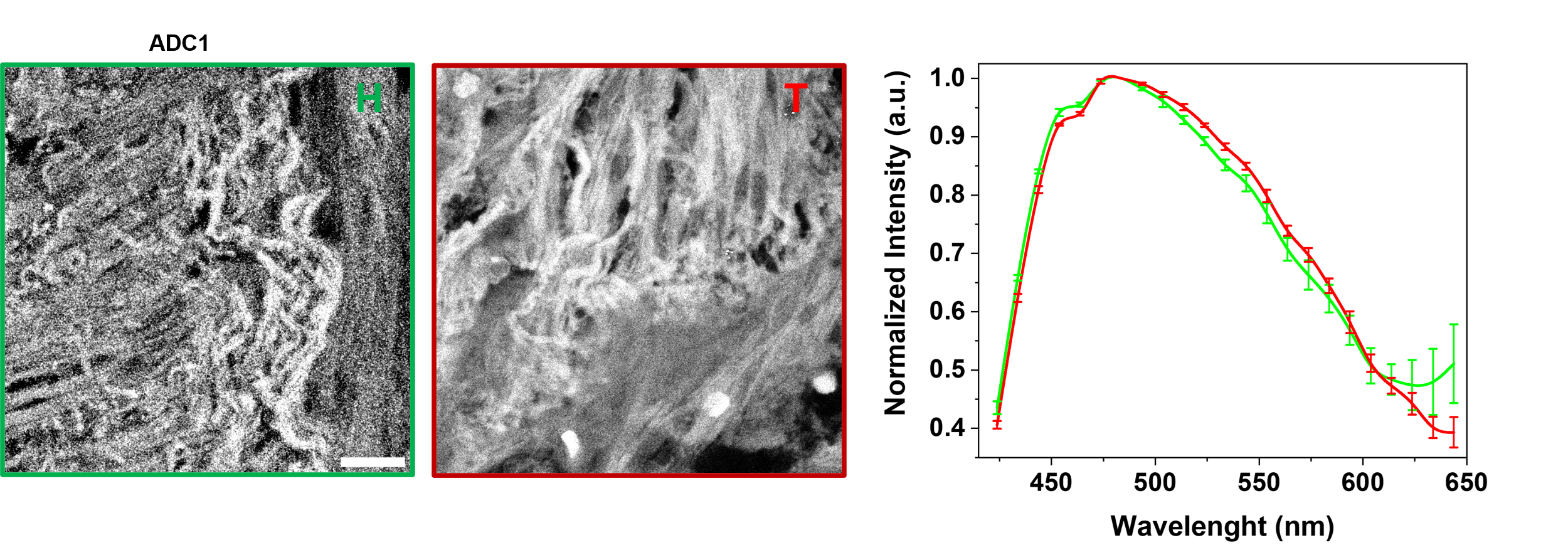


Figure S5. Fluorescence spectra of fluorescence fiber using 810nm infrared laser power. Scale bar 100µm.


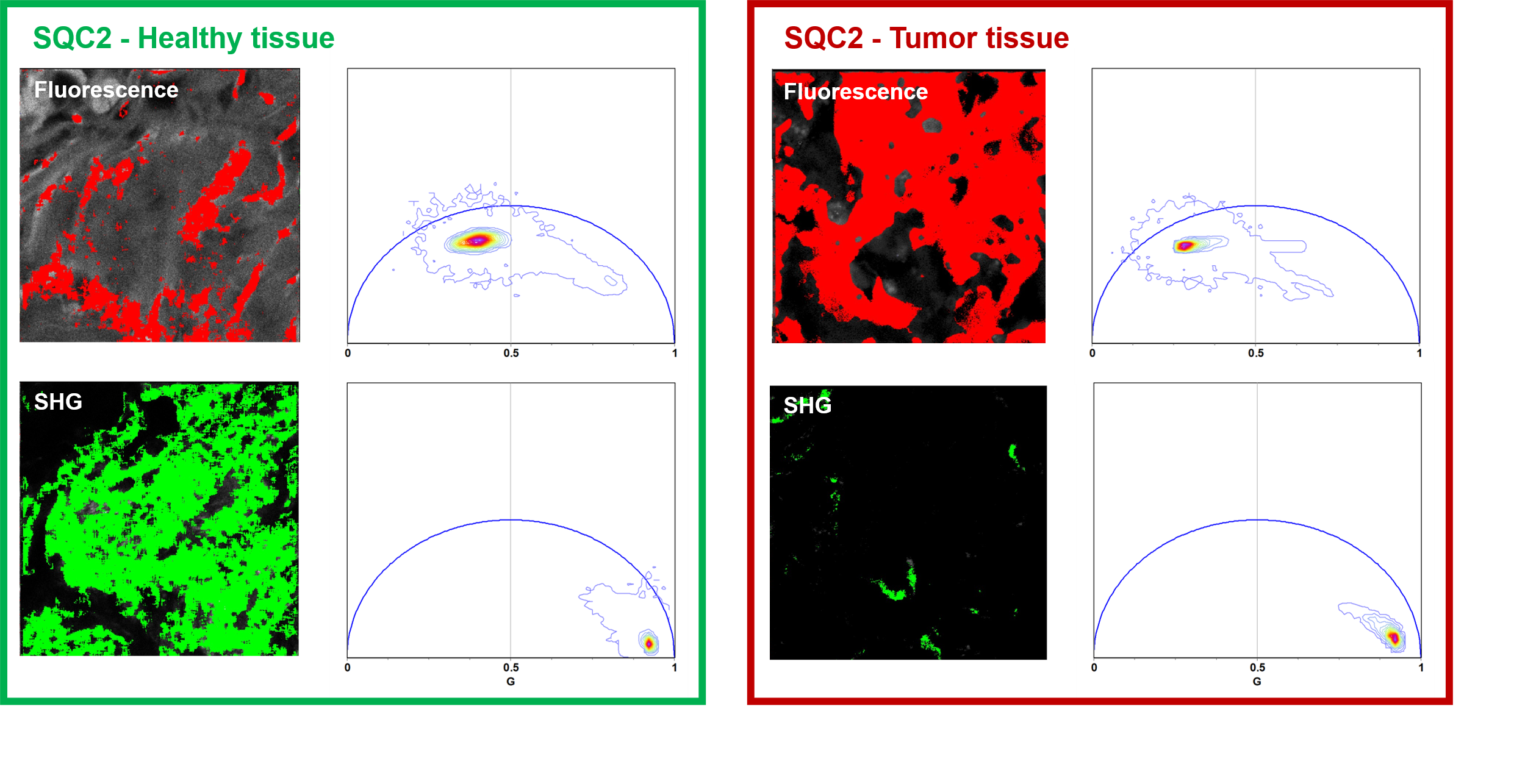

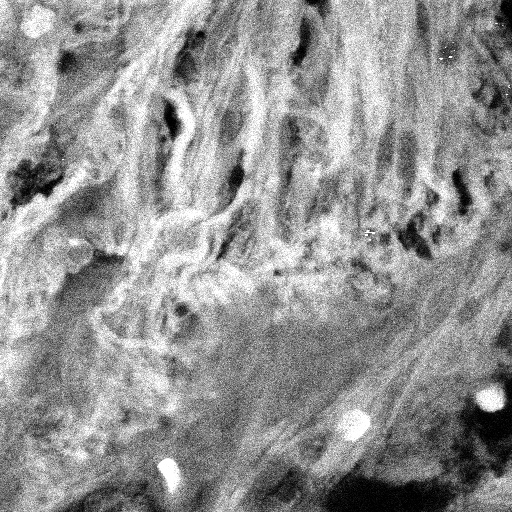


**H**

**T**

Figure S6. Comparison between healthy and tumor regions using phasor-FLIM analysis. Fluorescence (in red): excitation at 810 nm, emission filter 550/90 nm; SHG (in green): excitation at 810 nm, emission filter 405/10 nm.


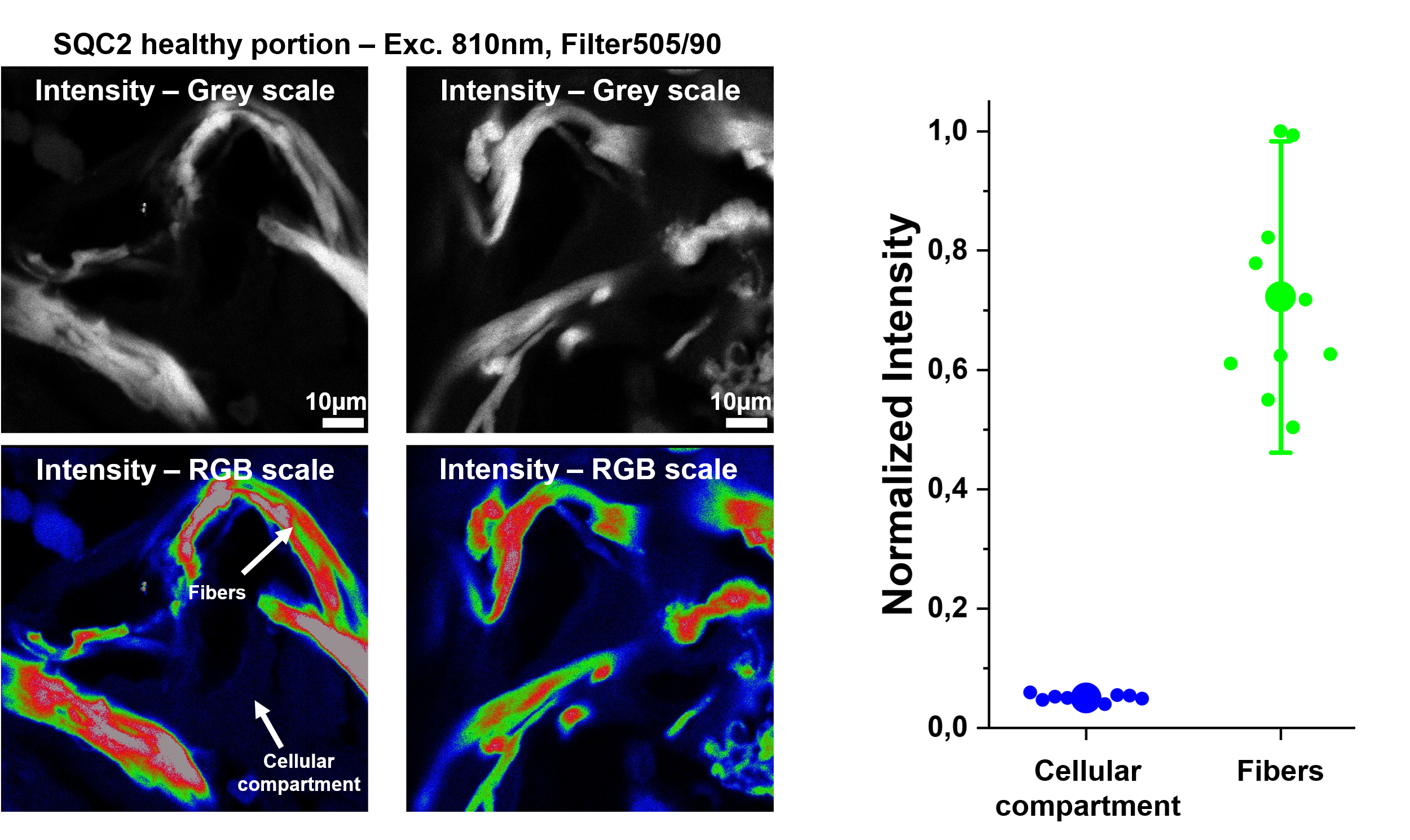


Figure S7. Characterization of the cellular compartment (FAD) at an excitation wavelength of 810 nm. An RGB LUT was applied to map pixel intensity values to corresponding colors. Values were normalized to the maximum value across all samples. Excitation: 810 nm; Emission filter: 505/90 nm.


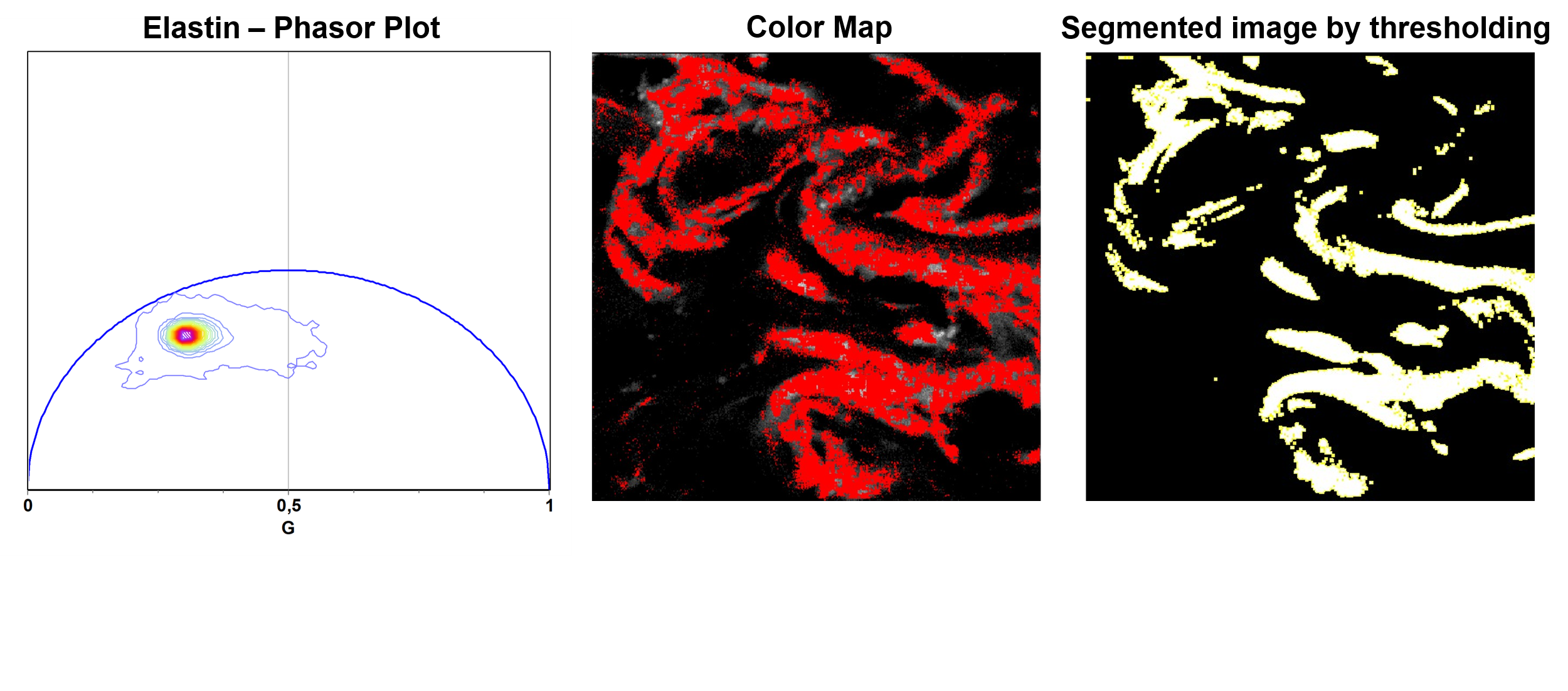


Figure S8. Thresholded and segmented elastin images in normal tissues. The segmentation resembles the color-map generated by selecting a specific lifetime in the phasor plot.


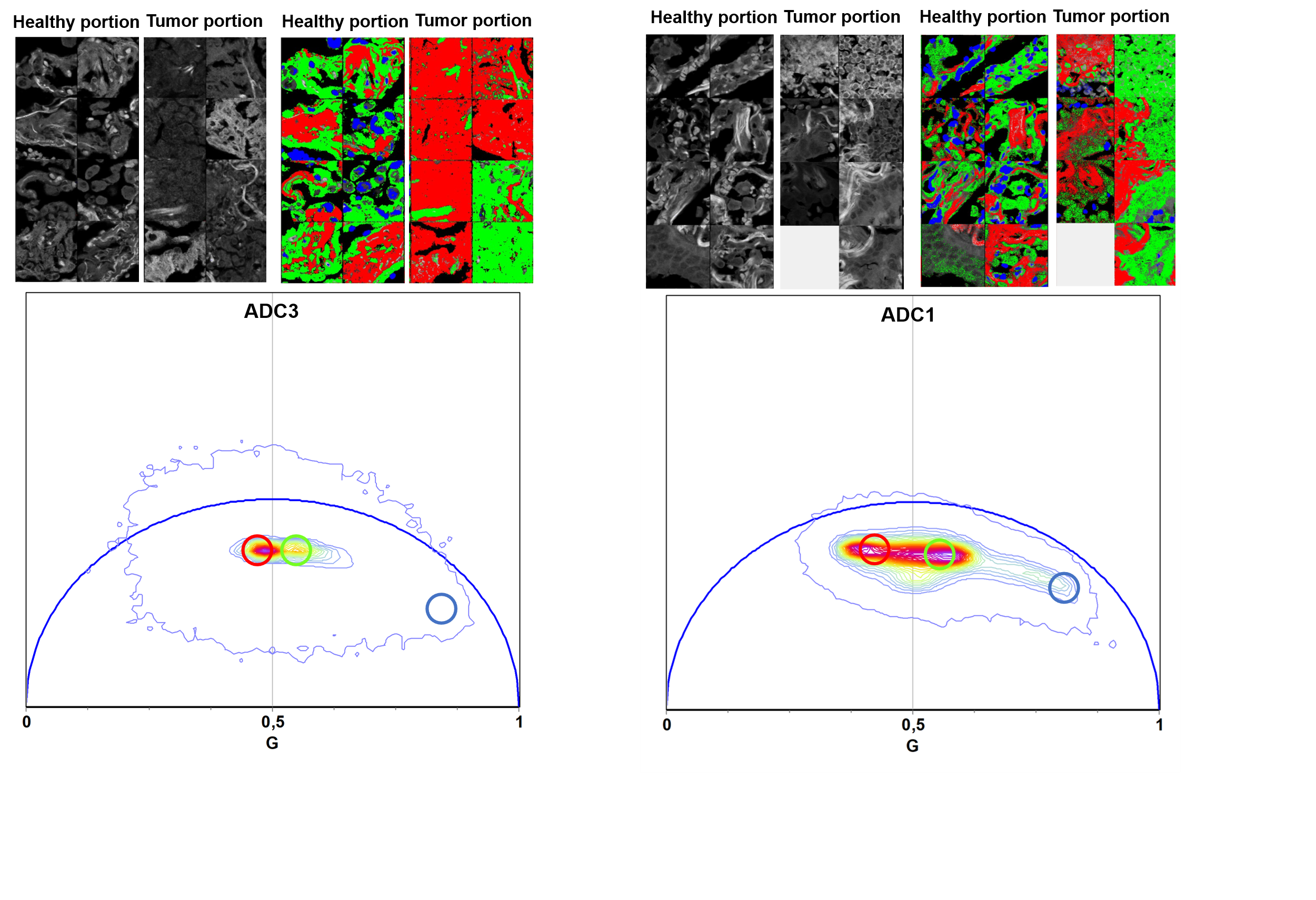


Figure S9. Colored map and optical signature of healthy and tumor regions in samples ADC3 and ADC1. Sample ADC3 shows differences in terms of cellular lifetime and red blood cells. In contrast, sample ADC1 shows no significant differences in the cellular compartment.


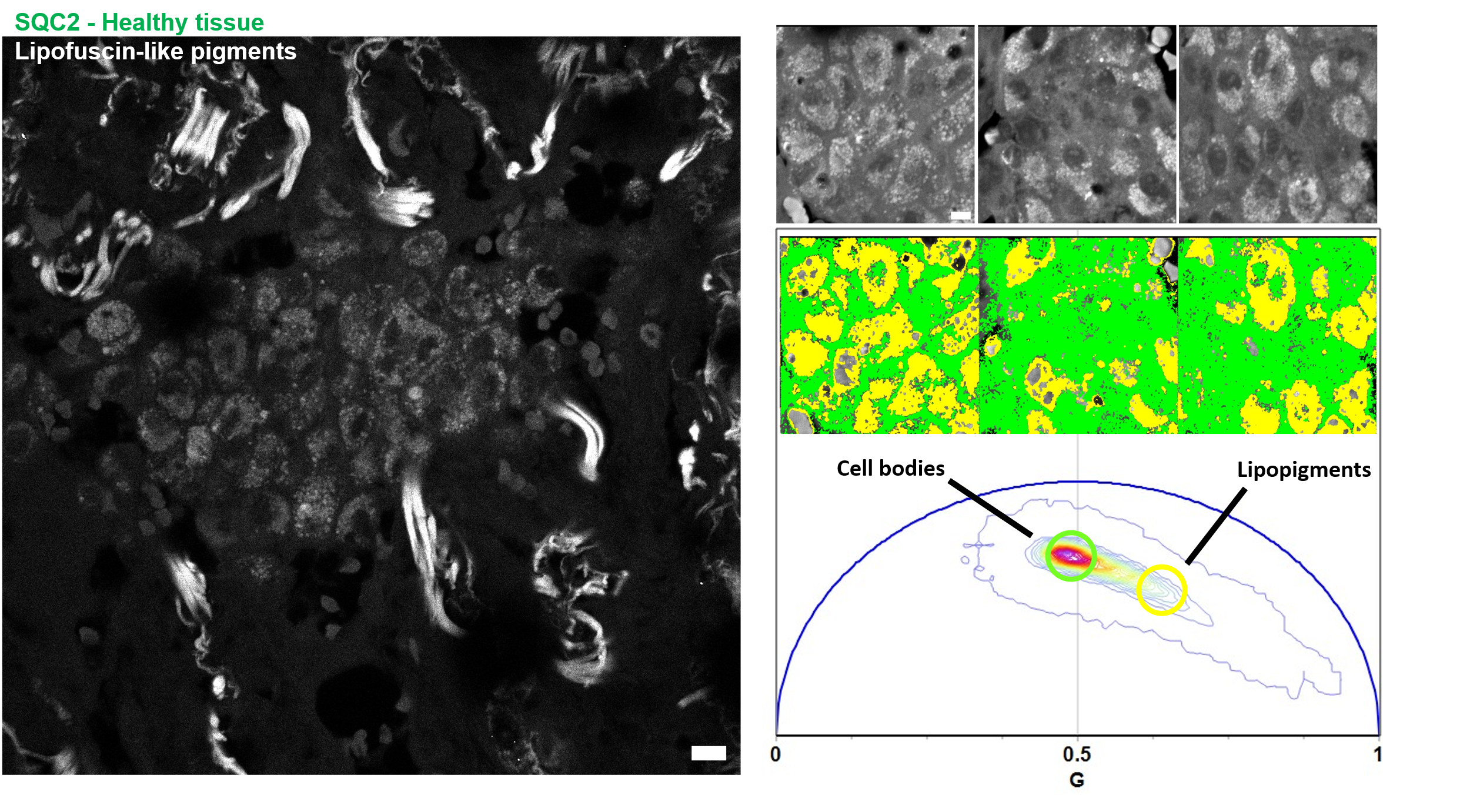


Figure S10. Imaging of a cluster of cells containing lipofuscin. Optical signature of lipofuscin-like accumulations (or ceroid) in healthy tissue. Excitation light 720nm; NAD(P)H filter 450/50. Scale bar 100µm.
